# Network Analysis of Internet Addiction, Online Social Anxiety, Fear of Missing Out, and Interpersonal Sensitivity among Chinese University Students

**DOI:** 10.1155/2024/5447802

**Published:** 2024-03-27

**Authors:** Xinyi Zhu, Wen Lian, Lu Fan

**Affiliations:** ^1^Department of Psychology, School of Education, Wenzhou University, Wenzhou, China; ^2^Department of Psychology, Jing Hengyi School of Education, Hangzhou Normal University, Hangzhou, China

## Abstract

**Background:**

Despite the growing prevalence of internet usage among young people, the relationships between internet addiction, online social anxiety, fear of missing out (FoMO), and interpersonal sensitivity remain uncertain, intricate, and multifaceted. To gain insight into the underlying psychological mechanisms, we employed network analysis to explore the interconnections between them. This endeavor may provide fresh opportunities for intervention and treatment.

**Methods:**

In this study, 470 participants were assessed at age from 18 to 22 (*M* = 20.18 years, SD = 1.861) years. Network analysis was used to examine the connections between symptoms, and statistical measures were applied to assess the stability of the network model.

**Results:**

Online social anxiety and interpersonal sensitivity had the strongest associations with other symptoms in the network, with “Evaluation anxiety” having the highest expected influence centrality, followed by “Privacy concern anxiety,” “Need for approval,” “Suspicion,” and “vulnerability.” The FoMO symptom, “Fear of missing information,” had the strongest direct relation to internet addiction. “Evaluation anxiety” and “Fear of missing information” played a key role in bridging internet addiction and interpersonal sensitivity. Additionally, the structure distribution of edge weights had a significant difference between gender.

**Conclusions:**

Our findings indicated that FoMO, interpersonal sensitivity, and online social anxiety likely play a significant role in the development and continuation of internet addiction. Interpersonal sensitivity seems to contribute to increased online social anxiety, FoMO, and the development of internet addiction, indicating that targeting these symptoms may help reduce negative online behavior and psychological burden.

## 1. Introduction

As the internet continues to rapidly develop and become more widely used, it is increasingly playing a crucial role in people's lives. Social media offers diverse activities, from connecting with loved ones to engaging in global movements, providing avenues for fostering positive mental health [1]. However, the accompanying problems cannot be ignored. The issue of internet addiction (IA) has gained recognition for its detrimental effects on individuals' overall well-being and mental health [2, 3]. Goldberg defined internet addiction disorder (IAD) as the negative impact of excessive internet use on an individual's physical and mental health, leading to impairment in social functioning when the person is unable to control their online time [4]. Although there is still some inconsistency in the terminology used to define IA (e.g., problematic internet use, pathological internet use; [2, 5, 6]), it is generally understood as a behavioral addiction characterized by core components such as salience, mood modification, tolerance, withdrawal, conflict, and relapse [7–9]. Prevalence estimates of IA in the general population are reported to be around 4% [10]. A 2020 Chinese Youth Internet Addiction Report found that 41.3% of teenagers use mobile phones to access the internet, with 15.2% of them classified as addicted [11]. Cross-sectional studies have established a connection between internet addiction (IA) and difficulties in interpersonal relationships, reduced well-being, academic challenges, and mental health issues such as anxiety and depression [12].

Frequent use of the internet, particularly social media, also exacerbates individuals' online social anxiety [13]. Online social anxiety refers to negative interpersonal experiences (e.g., tension, fear, and anxiety) generated by individuals in online social media communication situations, including privacy concern anxiety, other's negative evaluation anxiety, and interpersonal communication anxiety [14]. In fact, a majority of university students with social anxiety (87.8%) prefer to establish social relations through online self-disclosure, making them a vulnerable group for online social anxiety [15]. Recent studies have found that individuals with social anxiety disorder (SAD) show more severe symptoms of internet addiction compared to those without SAD [16–18], indicating a greater problem with internet use in this population. However, some researchers believe that individuals with SA are at a higher risk of experiencing internet-related issues, but the internet can also alleviate their anxiety in real-world communication [15, 19]. While both types of social anxiety involve complex mechanisms, they differ due to the unique nature of online social interaction [20]. The decompensation theory suggests that characteristics of online social interaction like anonymity and randomness lead to internet dependence, seen as anticipation, passion, and fear of rejection [21]. This dependence becomes a pathological compensation in psychological development, causing discomfort with negative information, tension in online interactions, and cognitive deviations, resulting in high online social anxiety [14]. Understanding these mechanisms in internet addiction (IA) is crucial for effective interventions.

Fear of missing out (FoMO) is considered a stable personality trait that is linked to the psychological mechanisms involved in internet addiction [22–26]. FoMO has been defined as a pervasive anxiety wherein one is concerned that others may be enjoying rewarding experiences from which they are excluded, leading to a desire to stay continuously connected with others' activities [25]. Previous studies have found that FoMO can lead to excessive or addictive use of social media, reducing opportunities for face-to-face interaction and negatively impacting interpersonal trust, thus affecting individual behavior and both physical and mental health [27–29]. In addition, FoMO has been identified as a mediator in the relationship between health anxiety, gaming disorder, and problematic smartphone use during the COVID-19 pandemic [30]. Recent network analysis research indicates that FoMO, along with gaming motivation, may increase the time spent gaming and contribute to gaming disorder [31, 32]. What's more, FoMO has been found to predict online social anxiety in individuals [33]. Individuals with high FoMO tend to be selective in sharing content, which hinders social interaction and increases online social anxiety. According to self-determination theory, FoMO, serving as both an internet-related cognitive bias and a specific personality trait, may intensify IA severity by fulfilling social needs [30, 34, 35]. Decompensation theory suggests that individuals experiencing FoMO may struggle to find “constructive compensation” in the network to compensate for developmental deficiencies. Consequently, they become excessively dependent on the network due to fears of missing out on information [21, 36]. It can be seen that FoMO can be a direct or indirect factor affecting internet addiction.

Interpersonal sensitivity (IS), regarded as an enduring personality trait, describes an individual's sensitivity towards the emotions and behaviors of others [37]. IS involves excessive attention to and sensitivity towards interpersonal relationships, social feedback, others' behavior and emotions, and criticism or rejection [38, 39]. Those with high levels of IS often experience feelings of inferiority, uneasiness, noticeable discomfort, and negative expectations regarding interpersonal communication [40]. IS is seen as crucial for social adaptation, as it involves accurately assessing others' abilities, characteristics, and emotions [41, 42]. This personality factor overlaps with several other individual difference variables related to physical health, such as introversion, compliance, social inhibition, rejection sensitivity, social avoidance, social anxiety, and type D personality [39]. Studies suggest that high IS is linked to increased social anxiety [43, 44] and a higher risk of developing addictions like internet and drug addiction [45]. Smartphone addiction research reveals a positive connection between IS, fear of missing out (FoMO), and smartphone addiction, with FoMO mediating IS and smartphone addiction [46]. However, further discussion is needed to determine the direct or indirect impact of interpersonal sensitivity on internet addiction.

Over the last two decades, there has been a rising trend in the application of network analysis (NA) across both social and physical sciences [47, 48]. From a network perspective, mental disorders are not underlying latent (unobservable) disease entities. Instead, they emerge as complex networks of mutually reinforcing symptoms [49, 50]. The advantage of NA lies in its graphic visualization, which provides a more intuitive depiction of the causal relationships between variables using graph theory. Through quantifying relationships among observed symptoms, network models can determine the most pivotal symptoms, known as “central symptoms.” Symptoms that exhibit significant associations between two disorders or communities are termed “bridge symptoms” [51]. These bridge symptoms play a crucial role in maintaining the network model by facilitating the transformation from one disorder to another, occurring simultaneously [52–54]. These symptoms can assist in identifying the underlying mechanisms responsible for the development and persistence of psychological issues, and thus targeting them could result in more efficient treatment strategies [55, 56].

Accordingly, network analysis is employed to investigate the following intricacies: firstly, to explore the relationship between IA, FoMO, online SA, and interpersonal sensitivity as separate groups of symptoms; secondly, to identify the key symptoms that play a central and influential role in the entire network; thirdly, to examine the potential connection between IA and interpersonal sensitivity, as well as the roles of FoMO and online SA as mechanisms of action; lastly, to compare the networks among university students of different genders. As this study is exploratory in nature, specific hypotheses were not formulated for the aforementioned objectives. In addition to pinpointing central and bridge symptoms, this study also concentrates on identifying symptoms directly associated with internet addiction, which can serve as a basis for developing interventions that specifically address university students' internet usage habits. It is crucial to provide university students with IA, online SA, FoMO, and interpersonal sensitivity with comprehensive guidance and targeted interventions to improve their mental health.

## 2. Methods

### 2.1. Participants and Study Procedure

An online survey was conducted with 529 university students from Zhejiang provinces in China. The study used a cross-sectional design and employed convenience sampling through a popular Chinese survey platform (http://www.sojump.com). Out of the original participants, 59 were excluded from the analysis due to their completion times that were either three standard deviations faster or slower than the average, or because they did not complete all survey items. The total sample of 470 participants comprised 145 male university students (mean age = 20.18, SD = 1.67) and 325 female university students (mean age = 20.11, SD = 1.94). The online survey took approximately 10 minutes to complete. All participants signed an online informed consent form before answering the online questionnaire. Personal information was not used in the network analysis, and the data were fully anonymized. The Ethics Committee of Wenzhou University approved this study.

### 2.2. Measures

The Chinese version of the Internet Addiction Scale (IAS) is an 8-item questionnaire, based on an abbreviated version of the self-administered Internet Addiction Test (IAT) developed by adapting DSM-IV criteria for pathological gambling to the internet use [57]. The original questionnaire was developed from a pathological perspective and answered with “yes” or “no.” The revised Chinese version of IAS uses a Likert rating of 5-point score (1 represents very disagree, 5 represents very agree), so these 8 items sum up to a total score ranging from 8 to 40, with a higher total score indicating severe IA status [58]. The developer of the IAS suggests that individuals with a total IAS score of 32–40 have significant problems related to internet use [59]. The IAS questions reflect several elements related to internet usage: for example, loss of control, withdrawal, preoccupation, social demeanor, sleep problems, and the decline in academic efficiency. The internal consistency of the IAS for university students' sample was Cronbach's alpha of 0.85.

Online social anxiety was measured by the Social Anxiety Scale for Social Media User (SAS-SMU; [60]), Chinese version [14]. The SAS-SMU has 20 items and scored on a Likert scale of 1 (completely disagree) to 5 (completely agree)—the total score ranges from 20 to 100 points. The three factors of the scale include “Evaluation Anxiety,” “Interaction Anxiety,” and “Privacy Concern Anxiety” (e.g., “I am worried that others may find my behavior awkward” and “My personal information may be shared publicly and make me feel anxious”; [14]). The Chinese version of the SAS-SMU has reliable internal consistency, with a Cronbach's alpha of 0.96 in this study.

The Interpersonal Sensitivity Measure (IPSM) was used to measure the sensitivity to interpersonal interactions, social feedback, and negative evaluations (actual or perceived) from others [38]. The Chinese version of the IPSM consists of five sub dimensions, namely, “Suspicion,” “Need for approval,” “Separation anxiety,” “Vulnerability,” and “Shyness” [61]. There are a total of 29 items, each of which is scored on a 4-point scale from 1 (completely disagree) to 4 (completely agree), with higher total scores indicating the higher level of interpersonal sensitivity. The Chinese version of the IPSM has been well validated in Chinese samples [61], with a Cronbach's alpha of 0.89 in this study.

The Fear of Missing Out Scale (FoMOS) is the most widely used scale to assess fear of missing out, including both online and offline contexts [25]. The Chinese version of FoMOS divides the missed objects into two categories, i.e., “fear of miss information” (e.g., “I am afraid that others have more wonderful experiences and gains than I”) and “fear of miss situation” (e.g., “When I miss the opportunity to meet friends, I feel annoyed”), with a total of 8 items [62]. Items are responded to on a five-point scale ranging from 1 (“totally disagree”) to 5 (“totally agree”). A higher total score indicates a greater level of FoMO. The reliability and validity of this scale have been supported by multiple studies among Chinese university students [63, 64]. The internal consistency of the FoMOS in the present study was Cronbach's alpha of 0.83.

### 2.3. Data Analysis

Descriptive statistics, effect size (Cohen's *d*), Cronbach's alpha, and correlation analysis (i.e., Pearson and Bayesian) were conducted utilizing JASP (Jeffrey's Amazing Statistics Program; version 0.17.2). R package was used to perform the network analysis and the network comparison test (NCT) on gender.

#### 2.3.1. Network Estimation

For data analysis, the R program [65] was used to conduct NA. Within a network model, nodes represent study variables, and edges represent correlation between two nodes, which comprise network system [66]. Least absolute shrinkage and selection operator (LASSO) and extended Bayesian information criteria (EBIC) were used to shrink edges in the network, and the tuning parameters were set to 0.5 so that the symptom network was sparser and easier to interpret [67]. R packages qgraph (version 1.9.5) and bootnet (version 1.5.1) were applied to estimate and illustrate the network model visually. Edge thickness and darkness indicate the association strength between nodes/variables. Green edges represented positive associations, and red edges represented negative relations. The centrality index describes the relationship between multiple nodes including betweenness, closeness, and strength [66].

The EBICglasso domain-level network includes the total scores of the IAS, FoMOS, SAS-SMU, and IPSM. The facet level of the network includes nodes IA, three facets of SAS-SMU, two facets of FoMO, and five facets of IPSM data. The item-level network includes all items of IAS, SAS-SMU, FoMO, and IPSM data.

#### 2.3.2. Centrality and Stability

In order to quantify the importance of each node in the network, the expected influence was calculated via R package qgraph [66]. This approach is more appropriate for networks that comprise both positive and negative edges compared to the traditional centrality index (i.e., node strength) [68]. Nodes having higher strength centrality and expected influence in the network mode were considered to be more important [69]. Bridge expected influence was calculated to identify bridge symptoms using the bridge function via R package networktools (version 1.5.0). Bridges having higher expected influence values reflected a greater risk of transferring from one community (i.e., variable) to other communities compared to bridges with lower expected influence values [54]. Moreover, R package mgm (version 1.2-13) was used to compute the predictability of each node, an index that reflects controllability of the network model [70]. Predictability values represented interconnectedness or the extent to which a node was associated with its neighboring nodes and was expressed as the area in the rings around each node in the layout of the network.

To assess the accuracy and stability of the observed network model, R package bootnet (version 1.5.1) was used based on 1000 bootstraps with 95% confidence intervals performed for each node. The correlation stability coefficient (CS coefficient) was calculated to indicate the centrality stability of node, which is preferable to at least 0.25 and better with more than 0.5 [71].

#### 2.3.3. Network Comparison

Considering gender differences in IA, online SA, FoMO, and interpersonal sensitivity [36, 72–74], network characteristic differences between participating men and women were examined. The network structure and global network strengths between gender were assessed using a Network Comparison Test (NCT) between the two within gender models, using R package Network Comparison Test (version 2.2.1) with 1000 permutations.

## 3. Results

### 3.1. Descriptive Statistics and Correlation Analyses

In Table 1, descriptive statistics of the total sample and comparison of the study variables between genders were shown (current sample: *N* = 470, 31% male). There were significant differences between genders for internet addiction (*t* = −2.376, *p* < 0.05, Cohen's *d* = −0.252), “Evaluation Anxiety” (*t* = −2.878, *p* < 0.01, Cohen's *d* = −0.287), “Privacy Concern Anxiety” (*t* = −3.148, *p* < 0.01, Cohen's *d* = −0.328), “Vulnerability” (*t* = −3.370, *p* < 0.001, Cohen's *d* = −0.337), and online SA (*t* = −2.583, *p* < 0.001, Cohen's *d* = −0.258). Additionally, except for “shyness,” online SA, FoMO, and other nodes of interpersonal sensitivity were all significantly and positively associated with IA (all *p* < 0.001). The Bayesian correlation showed that all of log (BF10) were more than 5, which further verified the statistically significant correlations between IA and other variables (Supplement, Table S1). The items of the four scales are displayed in Supplementary Table S2. The Cronbach's alpha values for internal consistency are given in the Measures section and Supplementary Table S3.

### 3.2. Network Structure

**Figure 1:**
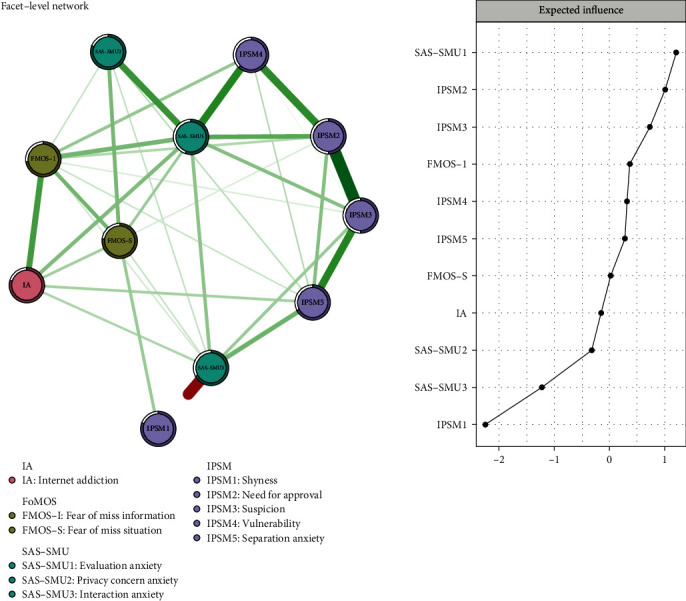
Network structure of facet-level model based on network analysis according to the relationships between IA, FoMO, online social anxiety, and interpersonal sensitivity in university students and the standardized estimates of node centrality. IAS: Internet Addiction Scale; FoMOS: Fear of Missing Out Scale; SAS-SMU: Social Anxiety Scale for Social Media User; IPSM: Interpersonal Sensitivity Measure.

Facet-level networks of internet addiction, fear of missing out, online social anxiety, and interpersonal sensitivity are depicted in the left panel of Figure 1. In terms of the fundamental traits of the facet-level networks, it is notable that 45 out of the 55 potential connections (56.1%) were active, indicating substantial interconnection among symptoms. Additionally, among the top ten most influential connections identified in the model, seven were situated within specific psychological trait communities (specifically, two within the online social anxiety community, four within interpersonal sensitivity, and one within the FoMO community).

In the facet-level network, nodes IPSM2 (“Need for approval”) and IPSM3 (“Suspicion”) had the strongest edge intensity (*r* = 0.464), while IPSM3 and IPSM5 (“Separation anxiety”) (*r* = 0.284), IPSM4 (“Vulnerability”), and SAS-SMU1 (“Evaluation anxiety”) (*r* = 0.278), as well as FoMO-I (“Fear of miss information”) and IA (*r* = 0.239), also had strong edge intensity (Figure 1, Supplement, Table S4). Node “Evaluation anxiety” had the highest level of strength centrality (1.623), followed by nodes IPSM2 (“Need for approval”) and IPSM3 (“Suspicion”) (1.27 and 0.719). Additionally, the nodes “Evaluation anxiety” also exhibited higher closeness (2.144) and betweenness (2.389) centrality (Supplement, Table S5). The CS coefficients of IA, FoMO, SAS-SMU, and IPSM were 0.61, 0.83, 0.87, and 0.78, respectively (Supplement, Figure S1).

Moreover, node predictability values ranged from 50.0% to 80.8% with an average of 67.5%, indicating that, on average, 67.5% of the variance in nodes from the network could be explained by their neighboring nodes (Figure 1 and Table 1). Online social anxiety symptom (“Privacy concern anxiety”) and internet addiction had the highest predictability in the model, while the interpersonal sensitivity symptoms, IPSM2 “Need for approval” and IPSM3 “Suspicion,” had the lowest predictability.

**Figure 2:**
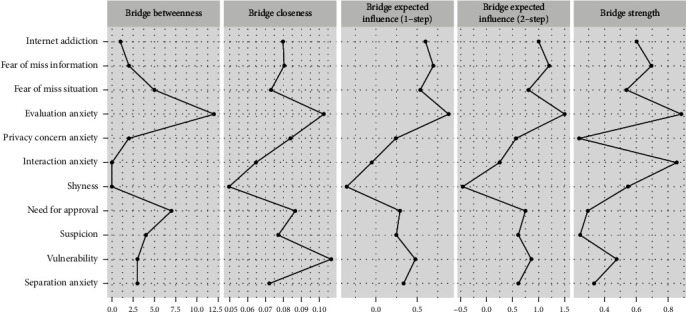
Bridge centrality plot of the facet-level network.

Expected influences within the entire network structure are shown in the right panel of Figure 1 and Table 1. SAS-SMU1 “Evaluation anxiety” had the highest expected influence value, followed by IPSM2 “Need for approval,” IPSM3 “Suspicion,” FoMO-I “Fear of miss information,” and IPSM4 “Vulnerability,” suggesting that these individual variables were the most influential within the entire network model in terms of variance explained. In contrast, other variables such as SAS-SMU3 “Interaction anxiety” and FoMO-S “Fear of miss situation” had a marginal impact within the network. Regarding bridge symptoms, “Evaluation anxiety” and “Fear of miss information” had the highest bridge expected influence values (Figure 2).

The EBICglasso domain-level network including IA, FoMO, SAS-SMU, and IPSM is shown in Supplement, Figure S2A. Nodes SAS-SMU and IPSM had the strongest edge intensity (*r* = 0.428). IA and FoMO had a strong edge intensity (*r* = 0.366). Nodes SAS-SMU and FoMO had a direct association with the other three nodes, respectively, while nodes IA and IPSM are not directly associated. The CS coefficients of IA, FoMO, SAS-SMU, and IPSM were 0.61, 0.83, 0.87, and 0.78, respectively. SAS-SMU (strength = 1.051, betweenness = 1.500, and closeness = 1.034) was identified as the most central node. The item level of the network including 8 items of IA, 20 items of SAS-SMU, 8 items of FoMO, and 29 items of IPSM data is shown in Supplement, Figure S2B. In the item-level network, nodes Ss6 (“I always feel that I will be criticized”) and PC2 (“My personal information may be publicly shared, which can make me feel anxious”) had a higher level of strength centrality (2.112 and 1.556) and expected influence (1.631 and 1.275), while node Ss5 (“If others know my true appearance, they will look down on me”) had the highest closeness (1.625), and IAn6 (“I feel nervous when I have to talk about myself with others”) had the highest betweenness centrality (2.273).

### 3.3. Network Stability

**Figure 3:**
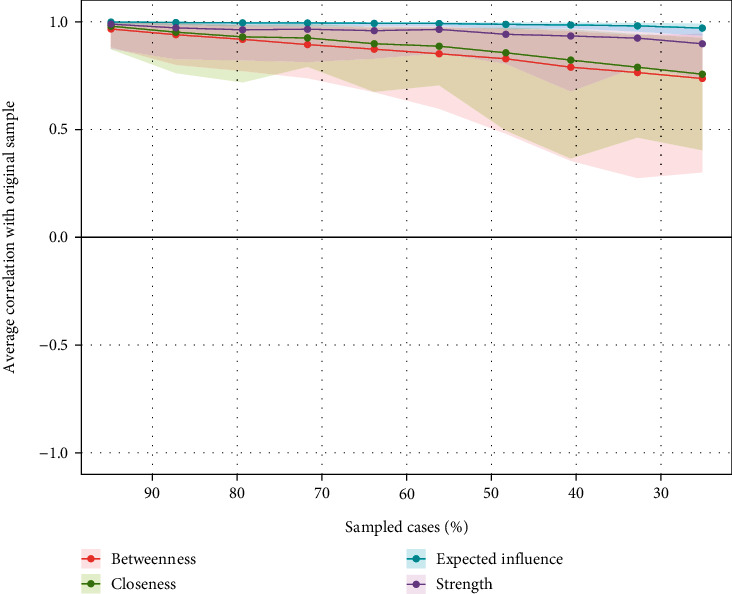
The stability of centrality indices by case dropping subset bootstrap.

As shown in Figure 3, both expected influence (EI) and strength centrality values indicated an excellent level of stability (both CS coefficients equaled 0.75), suggesting that 75% of participants could be dropped from analyses without significantly changing the network structure [69]. As measured by nonparametric CIs, the precision of edges was found to be satisfactory, with lower CIs suggesting more accurate edge estimates (Supplement, Figure S3). A considerable number of edge weight comparisons were statistically significant, according to the bootstrapped difference tests (Supplement, Figure S4).

### 3.4. Network Comparisons of Women versus Men

**Figure 4:**
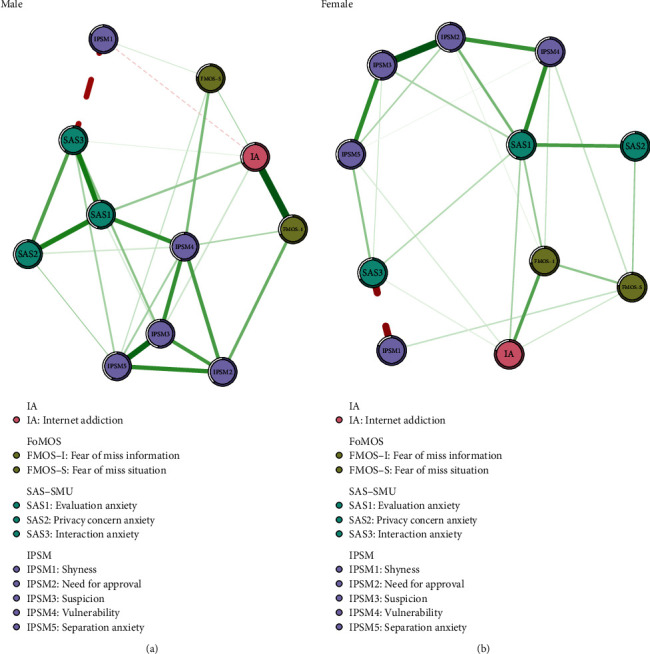
Facet-level EBICglasso model based on network analysis according to the relationships between IA, FoMO, online social anxiety, and interpersonal sensitivity among (a) males and (b) females.

The facet-level network structures generated from the two genders are shown in Figure 4. The structure distribution of edge weights had a significant difference between genders according to the Network Comparison Test (NCT) (*M* = 0.260, *p* = 0.047). But the global strengths showed no significant difference between males and females (4.596 vs. 4.795; *S* = 0.198, *p* = 0.581). Compared with males, females showed stronger associations between “Need for approval” and “Suspicion,” while internet addiction was strongly associated with “Fear of missing information” in males (Supplement, Figure S5).

## 4. Discussion

This study was the first to document the network structure of internet addiction, fear of missing out, online social anxiety, and interpersonal sensitivity among Chinese university students. Within this network, we discovered the strongest connections within specific psychological characteristic communities rather than between internet addiction and other variables. The most robust connection within the entire network was between two symptoms of interpersonal sensitivity: IPSM2 “Need for approval” and IPSM3 “Suspicion.” “Need for approval” refers to the desire for recognition and praise from others, as well as the tendency to prioritize their opinions and comply with their wishes in order to gain recognition [61, 75]. This symptom reflects how much individuals are willing to sacrifice their own needs to avoid humiliation or rejection and maintain the satisfaction of others, reflecting the motivational component of IS [61, 76], while “Suspicion” entails self-doubt, a lack of self-love, and a tendency to view others as unfriendly and untrustworthy [61]. The university student community is characterized by a desire for recognition from others [77]. On the other hand, the unique nature of the internet has amplified the anonymity and deceitfulness of online social activities [21]. The pursuit of recognition by individuals naturally leads to suspicion and distrust of others. Therefore, the “Need for approval” and “Suspicion” symptoms hold great significance within this network, and the strong positive correlation between them aligns with the characteristics of university students and the social dynamics shaped by the internet.

The second strongest connection identified in our sample was between SAS-SMU1 “Evaluation anxiety” and IPSM4 “Vulnerability.” “Evaluation anxiety” refers to the social anxiety that arises from how a person perceives and evaluates themselves based on others' opinions on social media platforms [60]. “Vulnerability” refers to the inability to handle criticism and negativity from others, often resulting in feelings of sadness and hurt [61]. It appears that both nodes contribute to monitoring an individual's fear of humiliation or rejection. Those who experience high levels of these symptoms often have fragile self-esteem, which relies on constant approval from others for reinforcement [76]. Feelings of inferiority and fragility can lead to anxiety and fear of negative judgments from others. However, having low expectations of others' evaluations can also make an individual feel even more inferior [72, 78]. Vulnerability is a way in which online social anxiety can manifest through interpersonal sensitivity.

Research has also revealed that one of the symptoms of FoMO, “Fear of missing information,” has the strongest direct relation to internet addiction. This finding aligns with a previous study that established a causal relationship between the fear of missing out and addiction to social media [79]. The psychological decompensation hypothesis suggests that excessive internet use serves as a compensatory mechanism for individuals encountering obstacles in their psychological development [36, 80]. Individuals who experience high levels of fear of missing out may develop an addiction to social media as a means of satisfying their psychological need to stay updated with the happenings around them [81]. This psychological comfort often elicits a sense of excitement in the individuals, leading to a loss of control and addiction during social media usage (it is worth noting that false beliefs may also contribute to this phenomenon) [79]. Based on the cognitive behavioral model, individuals with a high FoMO may initially develop irrational thoughts, believing that consuming social media content can alleviate their fear of missing out. As a result, they tend to rely on social media, which further reinforces their addiction during the satisfaction process [6]. In light of these findings, prevention and intervention by mental health professionals from any perspective can help break this harmful cycle and foster healthier development among university students.

The study found an unexpected negative correlation between the node IPSM1 “Shyness” and the node SAS-SMU3 “Interaction anxiety.” “Shyness” is defined as a tendency to be passive in communication and lacking confidence in expressing oneself [61]. On the other hand, “Interaction anxiety” refers to the social anxiety experienced during interactions, especially with new acquaintances on social media platforms [60]. Traditionally, it was thought that shy individuals would experience more anxiety in interpersonal relationships, while those who are outgoing and capable of making new friends would not exhibit shyness [82, 83]. However, this study suggests that shyness as a personal trait may not be linked to specific social situations [84], whereas social anxiety tends to be context-dependent. Additionally, the unique nature of the internet may reduce the burden of social anxiety for shy individuals, resulting in reduced levels of online social anxiety [85]. From this perspective, the results also partially indicate the benefits of internet use for some students (such as alleviating interaction anxiety), especially for those who exhibit shyness. However, as a significant medium in developing internet addiction, excessive immersion in the online world can also bring about many new issues. Other strong edges in the current network frequently existed between IS and online SA symptoms, suggesting that psychological factors in this network are noteworthy among university students and should be routinely evaluated in school referrals for mental health services.

The expected influence (EI) centrality of nodes performed well in identifying the specific symptoms that had the greatest impact on the overall network in this sample. One symptom, in particular, SAS-SMU1 “Evaluation anxiety,” had the highest centrality value, suggesting that it plays a crucial role in maintaining the entire symptom network. Consequently, interventions aimed at reducing excessive attention and expected anxiety towards negative evaluations from others have the potential to alleviate other symptoms in the network [86]. The fear of others' evaluations is a common feature of online social anxiety [60]. While there is limited research on the correlation between specific online social anxiety symptoms, it is important to note that recent empirical studies have found that online social anxiety plays a significant mediating and regulating role in models related to variables such as internet addiction and fear of missing out [87].

In addition to online SA symptoms, IPSM2 “Need for approval” and IPSM3 “Suspicion” were identified as central symptoms, partly due to their strong connection with each node of online social anxiety, which also showed that a series of adverse emotional problems caused by interpersonal sensitivity were very common among university students. Other central symptoms, including FoMO-I “Fear of miss information,” IPSM4 “Vulnerability,” and IPSM5 “Separation anxiety,” were also identified. Addressing these psychological symptoms through interventions may effectively reduce the severity of associated symptoms throughout the entire network. All of the identified central symptoms demonstrated high predictability values, accounting for 67.5% of the variance in average predictability for all nodes in the network. This indicates that a significant portion of the variation in nodes within the network can be explained by overlaps with neighboring nodes. Therefore, the symptoms examined in this study hold great significance in understanding the overall status of internet addiction among university students and can provide theoretical support for identifying the causes of internet addiction and developing intervention plans tailored to this population.

The study found that online social anxiety and fear of missing out were the top bridge symptoms in the network. The nodes “Evaluation anxiety” and “Fear of miss information” played a crucial role in connecting different nodes in the network. “Evaluation anxiety” was centrally positioned and connected to multiple other nodes, making it a key bridging variable. It was found that “Evaluation anxiety” serves as a bridge between IPSM4 and “Fear of miss information,” especially within the female group network. “Fear of miss information” was closely related to internet addiction and played a vital role in understanding the connection between internet addiction and other variables. It was also found that “Fear of miss information” had a stronger connection with internet addiction compared to online social anxiety, indicating that it has a more significant mediating effect on the relationship between internet addiction and other variables. However, further validation with a larger sample size is needed. Overall, the findings suggest that bridge symptoms in online social anxiety and fear of missing out communities can potentially exacerbate internet addiction and interpersonal sensitivity.

The study also found that female university students had higher scores in internet addiction, online social anxiety, “evaluation anxiety,” “privacy anxiety,” and “vulnerability” compared to male students, contradicting previous studies that found males generally scored higher in internet addiction [88]. However, other studies suggest that this difference may be related to specific types of internet addiction. For example, the majority of gaming addicts are male, while social media addiction is more prevalent among female addicts [8, 89, 90]. Some researchers argue that women may conceal negative emotions like anxiety and depression, leading to online addiction as a way to compensate for loneliness and social anxiety [91, 92]. The study also found that females had stronger connections between “Need for approval” and “Suspicion,” suggesting a higher need for online validation and cautious social interactions.

Network analysis has been used to examine various mental disorders, including depression, anxiety, psychosis, autism spectrum disorders, substance use disorders, posttraumatic stress disorder, insomnia, and other mental health symptoms and behaviors [93–98]. The focus of this study on online social anxiety, interpersonal sensitivity, and fear of missing out is all indicative of a range of psychological issues, including anxiety and depression, even though our study did not specifically address the relationship between social network use and other common psychological health issues like depression. Previous studies suggest that a variety of intricate mechanisms are involved in how social network use affects an individual's mental health. Negative emotions like anxiety and depression may be triggered by factors commonly associated with online social issues, including social comparison and self-evaluation, social pressures and loneliness, information overload, and exposure to negative content [99–102]. Additionally, avoiding face-to-face social interactions, sleep disorders brought on by unpleasant emotions, and excessive use of social media (also known as internet addiction) can all contribute to the indirect exacerbation of a variety of psychological and physical symptoms [103, 104]. According to our network research, specific aspects of certain personality traits, such as the need for approval from others and the fear of missing out on information, exacerbate people's feelings of uncertainty and anxiety in virtual social environments, especially impacting the mental health of heavy internet users [105, 106]. Analyzing the relationship between these factors and internet addiction not only aids in the development of more effective intervention strategies to promote healthier and more balanced online social behaviors, but it also sheds light on the potential role that social network use may play in the emergence and progression of individual mental health issues, particularly symptoms of anxiety or depression triggered by internet-related problems.

This study was the first NA study to investigate online social anxiety, fear of missing out, interpersonal sensitivity, and internet addiction symptoms among Chinese university students. Aside from its unique focus, this study utilized an innovative network analysis perspective in its research methods, providing valuable insights into the sample of university students as well as differences between genders. However, it is important to acknowledge some limitations. Firstly, the study design was cross-sectional, which means that causal relationships between symptoms over time could not be determined. Secondly, for the sake of convenience in data collection and considering the impact of the number of factors on the process of network analysis, we opted to use only the abbreviated version of the Internet Addiction Scale (IAS) to measuring IA, rather than the original scale. This may potentially reduce the validity of our assessment of internet addiction. For future studies, researchers might consider using more comprehensive scales to enrich studies in this field. Lastly, in our study, we did not consider individuals' performance on specific social networking platforms, which is highly associated with internet addiction and online social anxiety. Subsequent research could employ more specific scales, such as the Facebook Intrusion Questionnaire and the Microblog/WeChat Network Social Behavior Scale, to further explore individuals' specific patterns of social network usage or detrimental habits within these networks.

In summary, the network model has highlighted symptoms associated with online SA and IPSM as being the most crucial among university students. It is important to focus on these specific symptoms when designing interventions to address common psychological problems and enhance healthy internet usage habits among students. Furthermore, interventions targeting FoMO symptoms can play a significant role in reducing internet addiction and online SA, as well as improving interpersonal sensitivity among Chinese university students.

## 5. Conclusion

A network analysis was conducted to examine the relationship between internet addiction (IA), interpersonal sensitivity, fear of missing out (FoMO), and online social anxiety. The study found a strong association between IA and online social anxiety, interpersonal sensitivity, and particularly FoMO, supporting the compensation theory of internet addiction. The results suggest that online social anxiety and interpersonal sensitivity are key factors in this network, and there were significant gender differences in the core symptoms. Interpersonal sensitivity appears to contribute to heightened online social anxiety, FoMO, and the onset of internet addiction, indicating that addressing these symptoms could potentially alleviate negative online behaviors and psychological distress.

## Figures and Tables

**Table 1:** Descriptive statistics of measurement variables and sample characteristics.

Variables	Total (*n* = 470)	Males (*n* = 145)	Females (*n* = 325)	*t*	*p*	Cohen's *d*	Expected influence^a^	Predictability^b^
Age (years)	20.39 ± 4.88	20.32 ± 1.68	20.42 ± 5.76	-0.200	0.842	-0.020	—	—
Internet addiction	23.19 ± 6.18	22.12 ± 6.83	23.67 ± 5.82	-2.526	0.012	-0.252	0.696	0.794
FoMO	24.78 ± 5.76	24.54 ± 5.82	24.89 ± 5.74	-0.598	0.550	-0.060	—	—
Fear of miss information	11.25 ± 3.74	11.15 ± 3.80	11.29 ± 3.72	-0.376	0.707	-0.038	0.793	0.724
Fear of miss situation	13.53 ± 2.97	13.39 ± 3.01	13.60 ± 2.95	-0.687	0.492	-0.069	0.774	0.769
SAS-SMU	64.66 ± 15.21	61.97 ± 16.45	65.87 ± 14.50	-2.583	0.010	-0.258	—	—
Evaluation anxiety	32.95 ± 8.30	31.32 ± 8.59	33.68 ± 8.07	-2.878	0.004	-0.287	1.280	0.553
Privacy concern anxiety	14.31 ± 3.95	13.43 ± 4.21	14.71 ± 3.77	-3.284	<0.001	-0.328	0.535	0.808
Interaction anxiety	17.40 ± 5.67	17.22 ± 5.70	17.47 ± 5.67	-0.446	0.655	-0.045	0.186	0.669
IPSM	73.93 ± 12.03	72.58 ± 12.09	74.53 ± 11.97	-1.626	0.105	-0.162	—	—
Shyness	16.86 ± 3.14	17.21 ± 3.22	16.70 ± 3.10	1.637	0.102	0.163	-0.313	0.802
Need for approval	12.38 ± 2.82	12.02 ± 2.77	12.54 ± 2.83	-1.842	0.066	-0.184	1.143	0.500
Suspicion	18.51 ± 4.99	18.31 ± 4.89	18.60 ± 5.05	-0.586	0.558	-0.059	0.925	0.515
Vulnerability	16.81 ± 3.51	16.01 ± 3.53	17.18 ± 3.45	-3.370	<0.001	-0.337	0.938	0.622
Separation anxiety	9.36 ± 2.56	9.03 ± 2.74	9.51 ± 2.46	-1.899	0.058	-0.190	1.003	0.670

^a^The values of expected influence were raw data generated from the facet-level network. ^b^The values of predictability were generated from the facet-level network.

## Data Availability

The data that support the findings of this study are available from the corresponding author upon reasonable request.

## Abbreviations

SA: Social anxiety
SAS-SMU: Social Anxiety Scale for Social media User
IA: Internet addiction
IAS: Internet Addiction Scale
FoMO: Fear of missing out
FoMOS: Fear of Missing Out Scale
FoMO-I: Fear of miss information
FoMO-S: Fear of miss situation
IS: Interpersonal sensitivity
IPSM: Interpersonal Sensitivity Measure
NA: Network analysis
EI: Expected influence
SAD: Social anxiety disorder
CS: Correlation stability
NCT: Network Comparison Test
CI: Confidence interval.

## Supplementary Materials

Supplementary Materials Table S1: correlation analysis of the study variables. Note: ∗∗*p* < .01, ∗∗∗*p* < .001. Table S2: items of IAS, SAS-SMU, FoMOS, and IPSM. Note: IAS, the Internet Addiction Scale; SAS-SMU, the Social Anxiety Scale for Social Media User; IPSM, the Interpersonal Sensitivity Measure; FoMOS, the Fear of Missing Out Scale. Table S3: reliability measures for network variable scales. Table S4: edge weight matrix of the facet-level network among total sample. Table S5: centrality measures per variable of the facet-level network among total sample. Figure S1: centrality stability (parametric) of the facet-level network among total sample. Figure S2: network structure of domain-level (A) and item-level (B) model based on network analysis according to the relationships between IA, FoMO, online social anxiety, and interpersonal sensitivity in university students. Figure S3: bootstrapped confidence intervals of edge weights. The analysis of the accuracy of edges, as implemented by means of nonparametric CIs, revealed that the precision of edges was acceptable, with smaller CIs indicating a more accurate estimation of edges. CIs for each population are small, which mean that the edge analysis is accurate. Figure S4: estimation of “edge” by bootstrapped difference test. The bootstrapped difference tests revealed that a large proportion of the comparisons among edge weights were statistically significant (black means significant, gray means not significant). Figure S5: standardized estimates of node centrality in the facet-level network between males and females' group.
