# Supplementary material for: Network Analysis of Internet Addiction, Online Social Anxiety, Fear of Missing Out, and Interpersonal Sensitivity among Chinese University Students
Source: Depress Anxiety. 2024 Mar 27;2024:5447802. doi: 10.1155/2024/5447802 (PMC11918617; doi:10.1155/2024/5447802)

**Supplementary materials**

Table S1.

|  | Internet addiction | FoMO-I | FoMO-S | Evaluation Anxiety | Privacy Concern Anxiety | Interaction Anxiety | Shyness | Need for approval | Suspicion | Vulnerability | Separation anxiety |
| --- | --- | --- | --- | --- | --- | --- | --- | --- | --- | --- | --- |
| Internet addiction | — |  |  |  |  |  |  |  |  |  |  |
| Log(BF₁₀) | — |  |  |  |  |  |  |  |  |  |  |
| FoMO-I | 0.540*** | — |  |  |  |  |  |  |  |  |  |
| Log(BF₁₀) | 55.117 | — |  |  |  |  |  |  |  |  |  |
| FoMO-S | 0.444*** | 0.440*** | — |  |  |  |  |  |  |  |  |
| Log(BF₁₀) | 34.140 | 33.469 | — |  |  |  |  |  |  |  |  |
| Evaluation Anxiety | 0.543*** | 0.535*** | 0.430*** | — |  |  |  |  |  |  |  |
| Log(BF₁₀) | 56.125 | 53.961 | 31.719 | — |  |  |  |  |  |  |  |
| Privacy Concern Anxiety | 0.413*** | 0.317*** | 0.300*** | 0.600*** | — |  |  |  |  |  |  |
| Log(BF₁₀) | 28.808 | 15.161 | 13.210 | 72.163 | — |  |  |  |  |  |  |
| Interaction Anxiety | 0.401*** | 0.363*** | 0.311*** | 0.560*** | 0.451*** | — |  |  |  |  |  |
| Log(BF₁₀) | 26.728 | 20.992 | 14.360 | 60.564 | 35.566 | — |  |  |  |  |  |
| Shyness | 0.043 | 0.100 | 0.234*** | 0.008 | -0.062 | -0.200*** | — |  |  |  |  |
| Log(BF₁₀) | -2.382 | -0.991 | 6.799 | -2.679 | -2.044 | 4.142 | — |  |  |  |  |
| Need for approval | 0.427*** | 0.533*** | 0.397*** | 0.657*** | 0.393*** | 0.449*** | 0.152** | — |  |  |  |
| Log(BF₁₀) | 31.053 | 53.377 | 26.094 | 92.419 | 25.473 | 35.140 | 1.227 | — |  |  |  |
| Suspicion | 0.422*** | 0.498*** | 0.234*** | 0.644*** | 0.453*** | 0.540*** | -0.056 | 0.743*** | — |  |  |
| Log(BF₁₀) | 30.248 | 45.246 | 6.778 | 87.334 | 35.881 | 55.313 | -2.165 | 132.037 | — |  |  |
| Vulnerability | 0.469*** | 0.543*** | 0.461*** | 0.713*** | 0.465*** | 0.385*** | 0.026 | 0.690*** | 0.628*** | — |  |
| Log(BF₁₀) | 39.009 | 55.934 | 37.398 | 116.403 | 38.175 | 24.282 | -2.580 | 105.725 | 81.499 | — |  |
| Separation anxiety | 0.477*** | 0.472*** | 0.334*** | 0.574*** | 0.434*** | 0.527*** | 0.064 | 0.634*** | 0.676*** | 0.583*** | — |
| Log(BF₁₀) | 40.646 | 39.617 | 17.206 | 64.517 | 32.442 | 52.064 | -2.007 | 83.553 | 99.798 | 67.138 | — |

Table S2.

| **Scale** | **Variable** | **Items** |
| --- | --- | --- |
| IAS | IAS1 | I always thinking about previous experiences or next time I go online |
|  | IAS2 | I feel that I need to spend more time online to be satisfied |
|  | IAS3 | Repeated attempts to control, reduce, or stop internet use, but failed? |
|  | IAS4 | Do you feel uneasy, depressed, or irritable when you reduce or stop using the internet? |
|  | IAS5 | Is the actual time spent online each time longer than planned? |
|  | IAS6 | Did it damage important interpersonal relationships or lose educational or job opportunities due to online use? |
|  | IAS7 | Have you ever lied to family, friends, or others to conceal your involvement in the internet? |
|  | IAS8 | Using the internet as a way to avoid problems or relieve negative emotions |
| SAS-SMU | Evaluation Anxiety | I'm worried that others may find my behavior awkward. |
|  |  | I'm worried that others will mock the content I share on social media platforms. |
|  |  | I'm worried that others may not like the content I share on social media platforms. |
|  |  | I'm afraid my closest friend won't support my behavior online. |
|  |  | I feel uncomfortable when my friends publicly express their dislike for the content I share on social media platforms. |
|  |  | I will worry that my actions will not receive support from others. |
|  |  | I will worry that my friends will evaluate the content I share on social media in front of others. |
|  |  | I feel anxious about leaving a negative impression on others. |
|  |  | I will worry that others will look down on me. |
|  |  | I feel anxious about not meeting others' expectations. |
|  | Privacy Concern Anxiety | The possibility of my personal information being accessed by others can make me feel anxious. |
|  |  | My personal information may be publicly shared, which can make me feel anxious. |
|  |  | I feel uncomfortable when my friends share my personal information with people I don't know. |
|  |  | I feel anxious about social media companies/institutions holding privacy about my personal life. |
|  | Interaction Anxiety | Talking to people I just met can make me feel anxious. |
|  |  | Talking to people who are not very familiar with me can make me feel nervous. |
|  |  | I feel uneasy when making new friends. |
|  |  | I feel nervous when communicating with people I first meet online. |
|  |  | I am afraid of communicating with others. |
|  |  | I feel nervous when I have to talk about myself with others. |
| IPSM | Shyness | When I come into contact with strangers, I feel uneasy. |
|  |  | When communicating with people, I always come up with interesting topics. |
|  |  | When interacting with people, I tend to adopt a proactive attitude. |
|  |  | I am able to confidently and firmly express my views and positions. |
|  |  | I am a sociable person. |
|  |  | I feel confident in interacting with people. |
|  | Need for approval | I always need recognition and praise from others. |
|  |  | I often feel that others are talking about me behind my back. |
|  |  | Only when others approve of me can I be confident that I have accomplished something well. |
|  |  | I am afraid that if I express my emotions, it will be unbearable to others. |
|  |  | My value largely depends on how others perceive me. |
|  | Suspicion | If someone says something insignificant, I will connect with myself. |
|  |  | If others know my true appearance, they won't like me. |
|  |  | I often feel sad to myself. |
|  |  | I often feel that others' eyes are unfriendly. |
|  |  | If others know my true appearance, they will look down on me. |
|  |  | I always feel criticized. |
|  |  | I am never sure if others are satisfied with me. |
|  |  | I don't think others can understand me. |
|  | Vulnerability | I always care if others have responded to me. |
|  |  | If someone criticizes something I have done, I will feel uncomfortable. |
|  |  | I am worried about how others will perceive me. |
|  |  | If people around me hold a negative attitude towards me, I will feel unhappy. |
|  |  | When others are angry with me, I feel hurt. |
|  |  | I care about how others feel about me. |
|  | Separation anxiety | Because I am afraid of losing, I avoid getting too close to the people around me. |
|  |  | I feel very sad when my good friend gets close to others. |
|  |  | I feel insecure when saying goodbye to others. |
|  |  | When I leave someone, I feel anxious. |
| FoMOS | FoMOS-I | I am afraid that others will have more exciting experiences and gains than me. |
|  |  | I am afraid that my friends will have more exciting experiences and gains than me. |
|  |  | When I find out that my friends are having a great time without me, I feel annoyed. |
|  |  | When I don't know what my friends are busy with, I feel anxious. |
|  | FoMOS-S | When I miss the opportunity to meet friends, I feel annoyed. |
|  |  | When I have fun, it is important for me to share what has happened online with everyone. |
|  |  | When I miss a planned party, I feel annoyed. |
|  |  | When I travel, I still keep a close eye on my friends' latest trends. |

Table S3.

| Network variable scale | Cronbach’s alpha |
| --- | --- |
| IAS | 0.86 |
| FoMOS | 0.83 |
| SAS-SMU | 0.96 |
| IPSM | 0.89 |

Table S4.

| **variable** | **IA** | **FoMO-I** | **FoMO-S** | **SAS1** | **SAS2** | **SAS3** | **IPSM1** | **IPSM2** | **IPSM3** | **IPSM4** | **IPSM5** |
| --- | --- | --- | --- | --- | --- | --- | --- | --- | --- | --- | --- |
| **IA** | 0.000 | 0.286 | 0.142 | 0.137 | 0.042 | 0.057 | -0.033 | 0.000 | 0.000 | 0.000 | 0.065 |
| **FoMO-I** | 0.286 | 0.000 | 0.193 | 0.114 | 0.000 | 0.000 | -0.032 | 0.095 | 0.050 | 0.065 | 0.024 |
| **FoMO-S** | 0.142 | 0.193 | 0.000 | 0.064 | 0.028 | 0.080 | 0.145 | 0.085 | -0.163 | 0.164 | 0.038 |
| **SAS1** | 0.137 | 0.114 | 0.064 | 0.000 | 0.315 | 0.202 | -0.031 | 0.134 | 0.077 | 0.268 | 0.000 |
| **SAS2** | 0.042 | 0.000 | 0.028 | 0.315 | 0.000 | 0.089 | -0.036 | 0.000 | 0.000 | 0.026 | 0.072 |
| **SAS3** | 0.057 | 0.000 | 0.080 | 0.202 | 0.089 | 0.000 | -0.444 | 0.000 | 0.103 | -0.084 | 0.182 |
| **IPSM1** | -0.033 | -0.032 | 0.145 | -0.031 | -0.036 | -0.444 | 0.000 | 0.064 | -0.031 | 0.000 | 0.084 |
| **IPSM2** | 0.000 | 0.095 | 0.085 | 0.134 | 0.000 | 0.000 | 0.064 | 0.000 | 0.414 | 0.217 | 0.134 |
| **IPSM3** | 0.000 | 0.050 | -0.163 | 0.077 | 0.000 | 0.103 | -0.031 | 0.414 | 0.000 | 0.177 | 0.299 |
| **IPSM4** | 0.000 | 0.065 | 0.164 | 0.268 | 0.026 | -0.084 | 0.000 | 0.217 | 0.177 | 0.000 | 0.105 |
| **IPSM5** | 0.065 | 0.024 | 0.038 | 0.000 | 0.072 | 0.182 | 0.084 | 0.134 | 0.299 | 0.105 | 0.000 |

Table S5.

| **variable** | **Betweenness** | **Closeness** | **Strength** | **Expected influence** |
| --- | --- | --- | --- | --- |
| Internet | 0.000 | 0.009 | 0.762 | 0.696 |
| Fear of miss information | 1.000 | 0.010 | 0.857 | 0.793 |
| Fear of miss situational | 6.000 | 0.012 | 1.101 | 0.774 |
| Evaluation Anxiety | 14.000 | 0.014 | 1.341 | 1.280 |
| Privacy Concern Anxiety | 0.000 | 0.010 | 0.607 | 0.535 |
| Interaction Anxiety | 8.000 | 0.012 | 1.241 | 0.186 |
| Shyness | 1.000 | 0.010 | 0.898 | -0.313 |
| Need for approval | 0.000 | 0.011 | 1.143 | 1.143 |
| Suspicion | 7.000 | 0.012 | 1.313 | 0.925 |
| Vulnerability | 4.000 | 0.013 | 1.105 | 0.938 |
| Separation anxiety | 4.000 | 0.011 | 1.003 | 1.003 |

Figure S1.


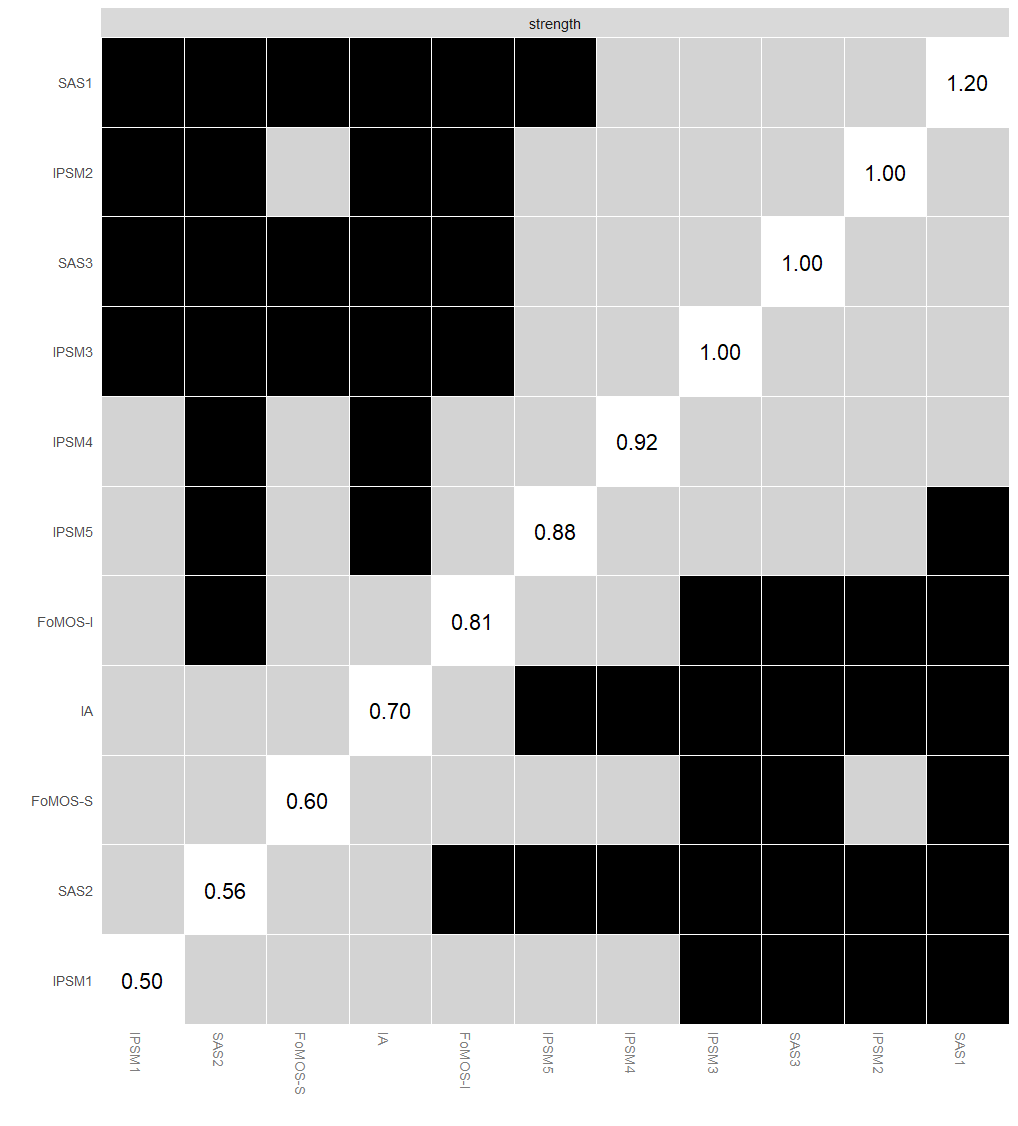


Figure S2.


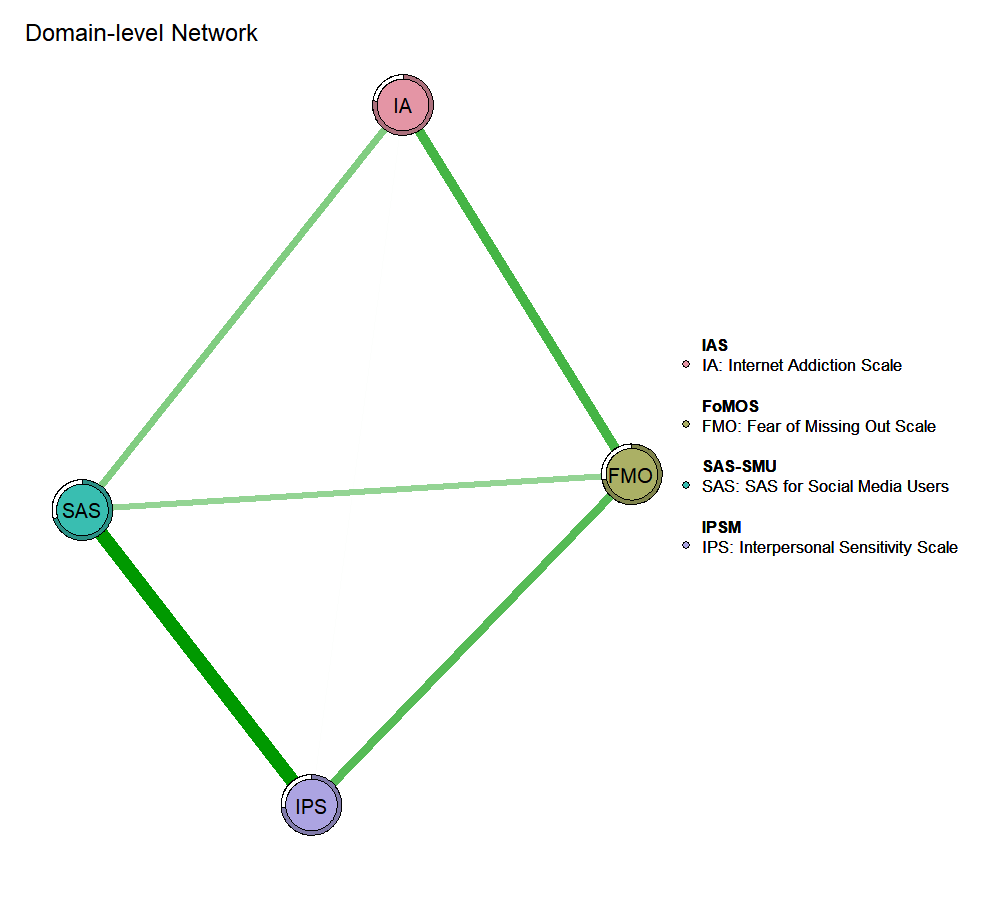

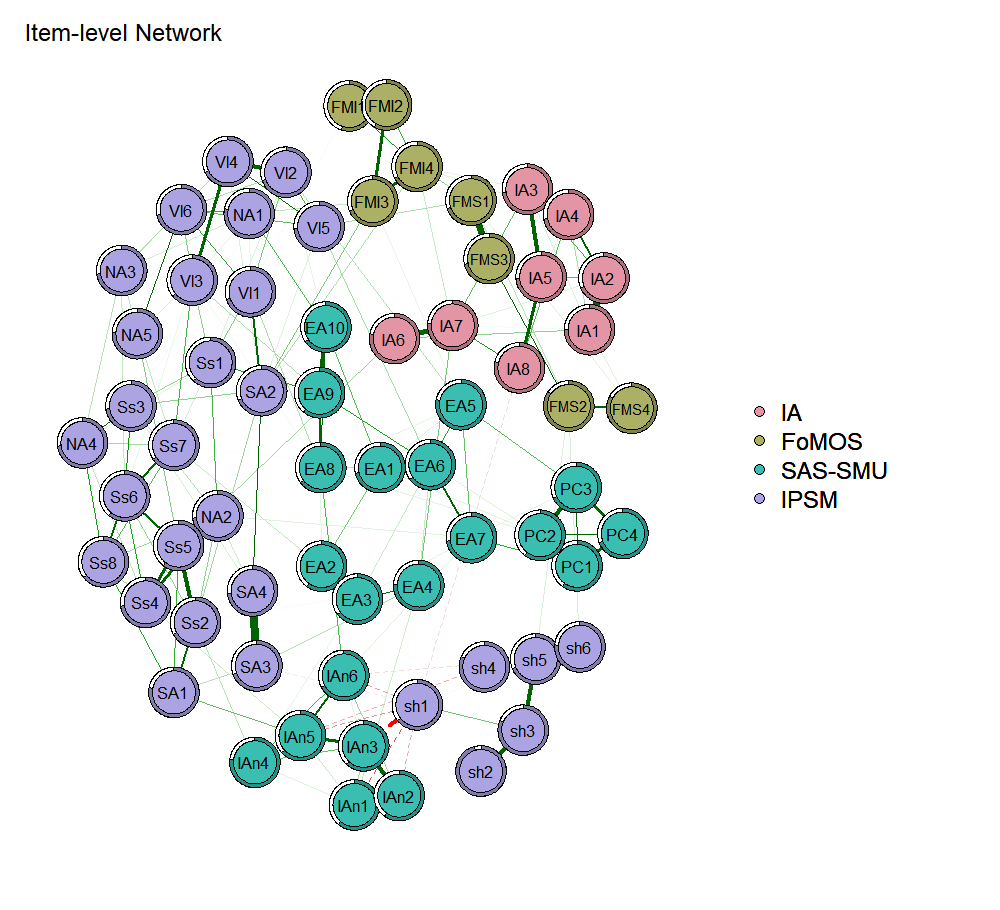


Figure S3.


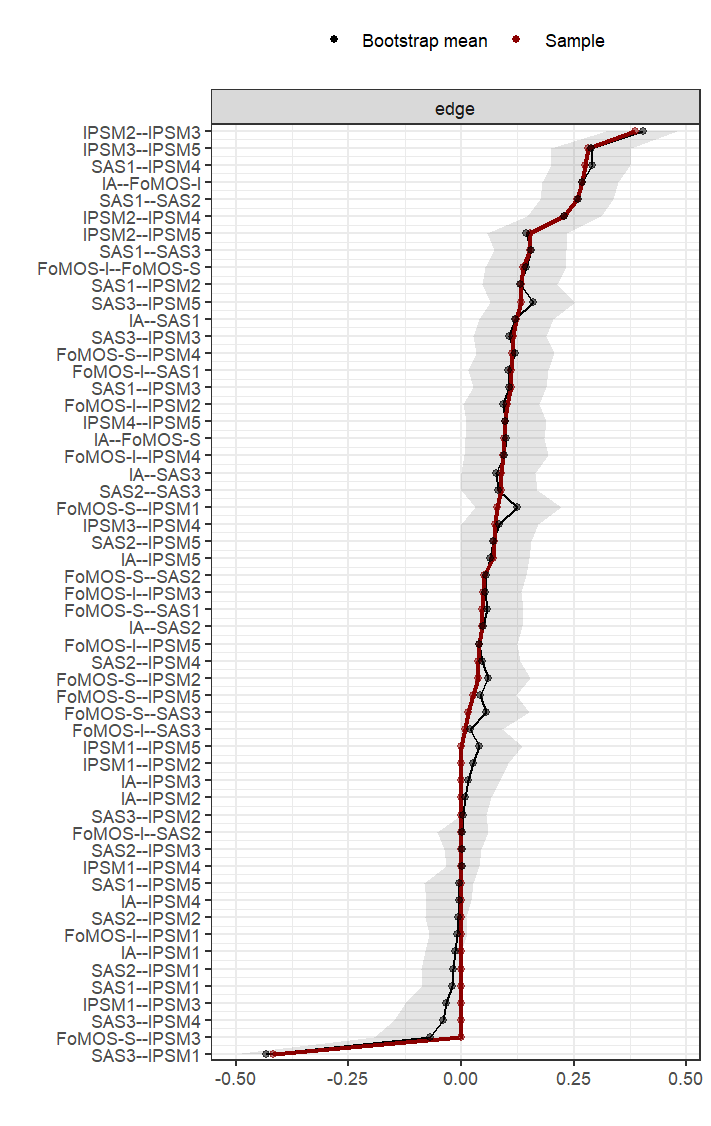


Figure S4.


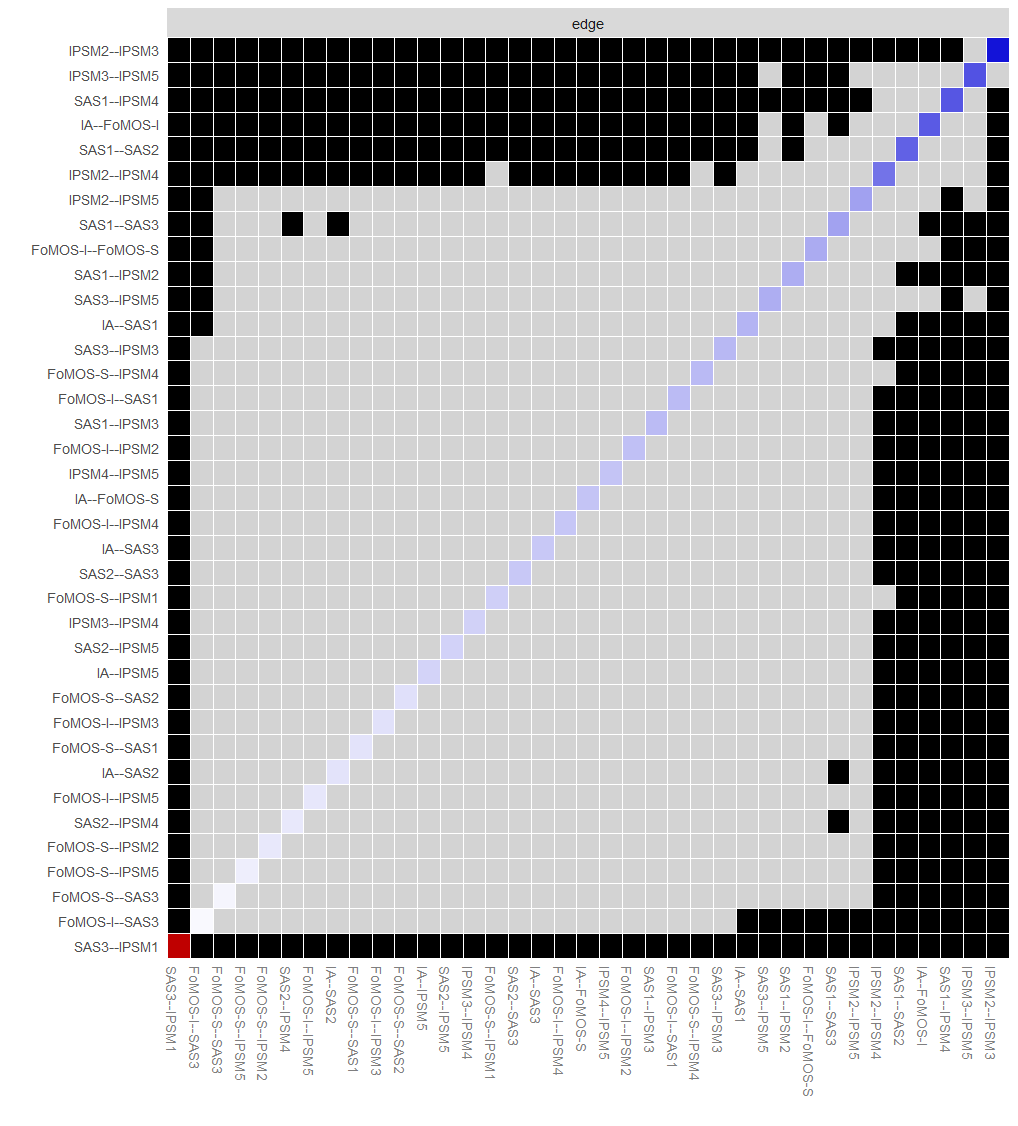


Figure S5.


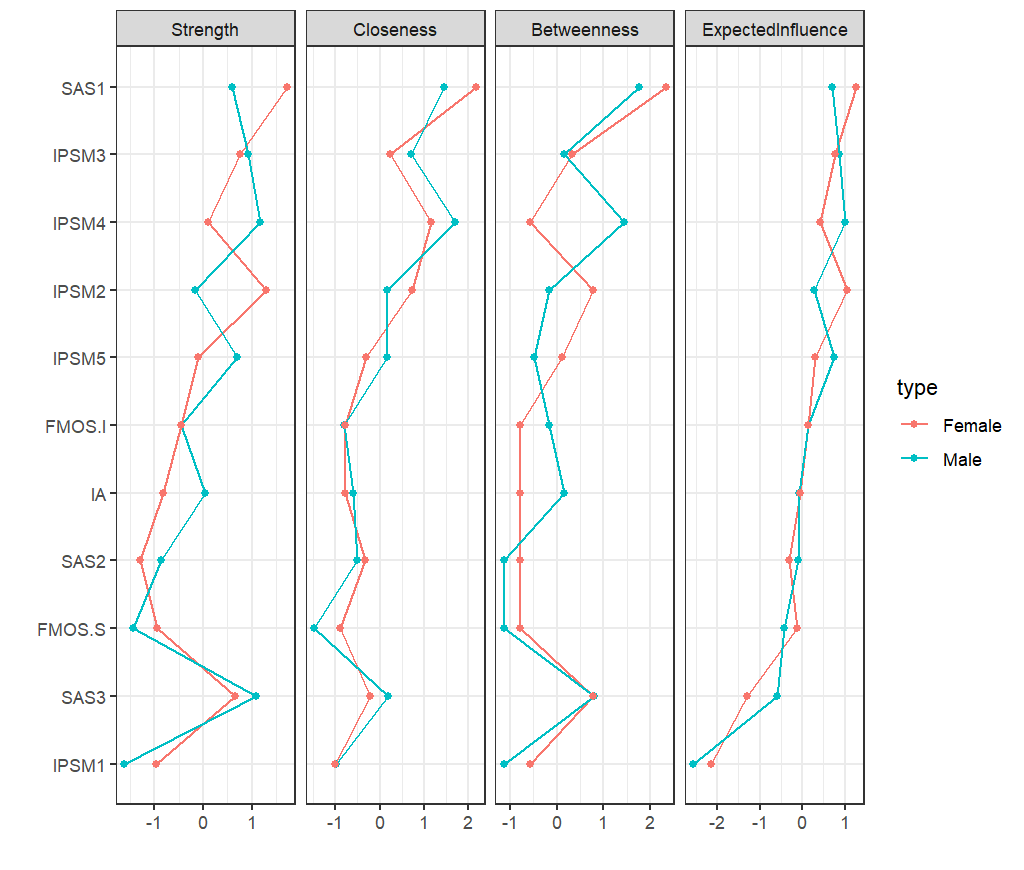

Supplement: Supplementary Materials — Table S1: correlation analysis of the study variables. Note: ∗∗p < .01, ∗∗∗p < .001. Table S2: items of IAS, SAS-SMU, FoMOS, and IPSM. Note: IAS, the Internet Addiction Scale; SAS-SMU, the Social Anxiety Scale for Social Media User; IPSM, the Interpersonal Sensitivity Measure; FoMOS, the Fear of Missing Out Scale. Table S3: reliability measures for network variable scales. Table S4: edge weight matrix of the facet-level network among total sample. Table S5: centrality measures per variable of the facet-level network among total sample. Figure S1: centrality stability (parametric) of the facet-level network among total sample. Figure S2: network structure of domain-level (A) and item-level (B) model based on network analysis according to the relationships between IA, FoMO, online social anxiety, and interpersonal sensitivity in university students. Figure S3: bootstrapped confidence intervals of edge weights. The analysis of the accuracy of edges, as implemented by means of nonparametric CIs, revealed that the precision of edges was acceptable, with smaller CIs indicating a more accurate estimation of edges. CIs for each population are small, which mean that the edge analysis is accurate. Figure S4: estimation of “edge” by bootstrapped difference test. The bootstrapped difference tests revealed that a large proportion of the comparisons among edge weights were statistically significant (black means significant, gray means not significant). Figure S5: standardized estimates of node centrality in the facet-level network between males and females' group. [file 5447802.f1.docx]
